# Twelve-year trends in cervical cytology screening: a retrospective analysis of 83,148 smears from a Turkish tertiary care center

**DOI:** 10.3389/fpubh.2026.1818289

**Published:** 2026-04-28

**Authors:** Berivan Guzelbag, Esen Gul Uzuner, Melik Selcuk, Selma Ermin, Cagseli Goksu Ozgun Selcuk, Numan Cim

**Affiliations:** 1 Department of Obstetrics and Gynecology, Haseki Training and Research Hospital, University of Health Sciences, Istanbul, Türkiye; 2 Department of Molecular and Medical Genetics, Institute of Graduate Studies, Biruni University, Istanbul, Türkiye; 3 Department of Pathology, Haseki Training and Research Hospital, University of Health Sciences, Istanbul, Türkiye

**Keywords:** cervical cancer screening, cervical cytology, HPV, HPV genotype, Pap smear, epidemiology, Türkiye

## Abstract

**Introduction:**

Cervical cancer screening through cytology and human papillomavirus (HPV) testing is essential for early detection of precancerous lesions, yet long-term institutional data integrating cytological trends, HPV genotype epidemiology, and diagnostic performance from Turkey remain limited. This study aimed to analyze the distribution and temporal trends of cervical cytological abnormalities, HPV prevalence and genotype distribution, and cytology-histopathology concordance over a 12-year period at a tertiary care center in Istanbul.

**Methods:**

A retrospective analysis of all cervical smear samples collected between January 2014 and December 2025 was conducted. Cytology results were classified according to the Bethesda 2014 system. HPV testing, which was performed on a subset of the study population based on clinical indications and kit availability, included mRNA-based assay (Aptima, 2020–2021, *n* = 4,648) and DNA genotyping (2024–2025, *n* = 4,308). Cytology-histopathology correlation was assessed for cases with biopsy within 180 days.

**Results:**

Among 83,148 cervical smear records, 76,232 evaluable results from women aged ≥21 years were included. The overall epithelial cell abnormality rate was 7.93%, with atypical squamous cells of undetermined significance (5.83%) and low-grade squamous intraepithelial lesions (1.77%) being the most common findings. Epithelial cell abnormality rates varied significantly across age groups, with the highest rate in the 18–20 age group (12.78%) and a significant inverse correlation with increasing age (*r* = −0.955, *p* < 0.001). Among tested patients, HPV positivity was 9.1% by mRNA testing and 9.9% by DNA genotyping, with HPV-16 predominating in both methods (20.5 and 32.8% of positive cases, respectively). Notably, HPV-56 (16.6%) and HPV-51 (14.3%) were more prevalent than HPV-18 (8.9%). High-grade squamous intraepithelial lesion cytology demonstrated a positive predictive value of 89.2% for detecting cervical intraepithelial neoplasia grade 2 or higher. A significant decrease in epithelial cell abnormality rates was observed over the study period (*r* = −0.591, *p* = 0.043), with a more pronounced decline in high-grade abnormalities (*r* = −0.911, *p* < 0.001).

**Conclusion:**

The 12-year analysis shows a decrease in cervical cytological abnormalities and provides valuable epidemiological data on HPV genotype distribution in a Turkish population. The high prevalence of non-vaccine-targeted genotypes, such as HPV-56 and HPV-51, should be considered in future vaccination strategies.

## 1 Introduction

Cervical cancer is a significant global health issue. According to estimates from 2022, there were 662,000 new cases and 349,000 deaths worldwide. Cervical cancer is the fourth most common malignancy among women (1). Approximately 85% of cases and 90% of deaths occur in low- and middle-income countries. This situation shows major differences in access to preventive services, screenings, and treatments (2, 3). From 1990 to 2019, age-standardized incidence rates declined in high-income countries with established screening programs. However, rates continued to rise in several regions of sub-Saharan Africa and Eastern Europe (4). The inverse relationship between cervical cancer incidence and the human development index highlights the importance of healthcare infrastructure and socioeconomic factors in disease prevention (2). Importantly, cervical cancer is one of the few major cancers that can be prevented with the methods that are currently available, which is why the World Health Organization (WHO) has called it the first cancer that can be eliminated as a public health problem (5).

The causal relationship between human papillomavirus (HPV) infection and cervical cancer was discovered by Harald zur Hausen. This discovery, for which he was awarded the Nobel Prize in Physiology or Medicine in 2008, is considered one of the most significant advances in cancer etiology (6). Almost all cervical cancers have been found to contain high-risk HPV subtypes, with HPV DNA identified in 99.7% of invasive cervical carcinomas around the world. HPV has been established as a necessary cause of this malignancy (7). The International Agency for Research on Cancer (IARC) classified 12 HPV genotypes (16, 18, 31, 33, 35, 39, 45, 51, 52, 56, 58, and 59) as Group 1 carcinogens, which means there is strong evidence that they cause cancer in humans (8). A comprehensive review of global research involving 111,902 cases of HPV-positive invasive cervical cancer from 121 countries identified 17 HPV genotypes related to cervical cancer development (9). HPV-16 has the highest worldwide percentage of cases that can be attributed to it (61.7%), followed by HPV-18 (15.3%), HPV-45 (4.8%), HPV-33 (3.8%), HPV-58 (3.5%), HPV-31 (2.8%), and HPV-52 (2.8%) (9). High-risk HPV infection that persists can progress to cervical intraepithelial neoplasia (CIN). CIN can progress to invasive carcinoma through the effect of the viral E6 and E7 oncoproteins, which inactivate the tumor suppressor proteins p53 and retinoblastoma (10, 11).

Cervical cancer is the 18th most common cancer among Turkish women. In 2022, there were approximately 2,600 new cases, and the age-standardized incidence rate was 4.8 per 100,000 (1). Due to the limited population coverage of cytology-based screening programs despite decades of implementation, Turkey transitioned to a national HPV-based cervical cancer screening program in 2014 (12). The program targets women aged 30–65 years, with screening intervals of every five years. HPV DNA testing is used as the primary screening modality, and cytology triage is used for HPV-positive cases (13). Among one million women screened, the initial results demonstrated an HPV positivity rate of 3.5%, with HPV-16 being the most prevalent genotype, followed by HPV-51, −31, −52, and −18 (13). This cost-effective, population-based approach utilizes centralized mega-laboratories for HPV testing and has achieved a four- to fivefold increase in screening coverage compared to the previous cytology-based program. Research from different regions of Turkey has shown HPV positivity rates ranging from 3.5 to 20%, highlighting differences based on geography and demographics (14, 15). As the largest city in Turkey, Istanbul receives migration from all regions of the country, creating a demographically diverse population that may serve as a representative setting for evaluating HPV prevalence patterns across different population groups.

Over the past eight decades, cervical cancer screening has evolved significantly since the introduction of the Papanicolaou (Pap) test. Dramatic reductions in cervical cancer incidence and mortality in countries with organized screening programs have been attributed to this screening modality (16). First introduced in 1988 and updated in 1991, 2001, and most recently in 2014, the Bethesda System has provided standardized terminology for reporting cervical cytology worldwide (17). In the 2014 update, a two-step classification system was introduced for squamous intraepithelial lesions (LSIL/HSIL), along with updated information on HPV-related cervical cancer (17). The transition from cytology-based to HPV-based screening represents a major advance in cervical cancer prevention, supported by four European randomized controlled trials demonstrating that HPV DNA testing offers superior sensitivity and higher negative predictive value compared to cytology (18). The American Cancer Society guidelines recommend HPV testing every five years as a screening method for women aged 25 to 65 (19). Recently, the HPV mRNA test has emerged as a new approach that detects E6/E7 oncogene expression, a marker of transforming infections. This assay demonstrates comparable sensitivity to DNA-based methods while offering higher specificity for high-grade cervical lesions, potentially reducing unnecessary colposcopy referrals (20). In 2020, the World Health Organization (WHO) introduced the Global Strategy to Accelerate the Elimination of Cervical Cancer and set the 90–70-90 targets to be achieved by 2030. These targets were for 90% of girls to receive the full course of HPV vaccination by age 15, 70% of women to undergo screening at least twice (at ages 35 and 45), and 90% of women diagnosed with cervical disease to receive appropriate treatment. However, regional disparities persist in vaccination coverage, screening participation, and timely access to treatment. Evaluating local screening data within this global strategy provides an opportunity to assess the real-world alignment with these targets (5).

Although national cervical cancer screening programs are well established, data reflecting center-based experiences remain limited. In particular, there are very few studies in single-center settings that simultaneously evaluate long-term cytological abnormalities, HPV genotype distribution, and changes in cytology-histopathology concordance. Understanding these data at the institutional level is necessary not only to monitor screening performance but also to identify regional differences in HPV prevalence and cytological outcomes. Additionally, it provides practical guidance for optimizing daily clinical decision-making and referral strategies.

As a result, we retrospectively analyzed 12 years of cervical screening data (January 2014–December 2025) from a tertiary hospital in Istanbul serving a socioeconomically and demographically varied population. Specifically, we evaluated the following: (1) the distribution and temporal trends of cervical cytological abnormalities, (2) the prevalence and genotype distribution of HPV infections; (3) cytology-histopathology concordance and diagnostic performance indicators; and (4) the relationship between specific cytological results and histologically confirmed cervical intraepithelial neoplasia grade 2 or higher (CIN2+). This study provides local and concrete information for the prevention of cervical cancer based on long-term data obtained from a center with a high number of patients. Additionally, it supports the current strategies implemented in Turkey in line with the World Health Organization’s goals to eliminate cervical cancer.

## 2 Materials and methods

### 2.1 Study design and ethical approval

This study is a retrospective study conducted at the Department of Obstetrics and Gynecology, Haseki Training and Research Hospital, Istanbul University of Health Sciences, Turkey. The hospital is a tertiary care center serving various urban populations in Istanbul. We reviewed all cervical smear tests performed between January 2014 and December 2025. The study was approved by the Haseki Training and Research Hospital Ethics Committee (approval number 287–2025, date December 10, 2025). Due to the retrospective nature of the study and the use of anonymized data, informed consent was not obtained.

### 2.2 Study population

Our study included all women who underwent cervical cytology screening over a 12-year period.

The primary population consisted of women aged 21 and older, in accordance with the American College of Obstetricians and Gynecologists (ACOG) guidelines recommending the initiation of cytology-based screening at age 21. Data from women aged 18–20 were also reported separately in age-stratified analyses to demonstrate trends across all age groups. This age group was excluded from routine testing recommendations due to the high rate of transient HPV infections and spontaneous regression of cervical abnormalities; therefore, these data were not included in the outcome calculations (overall ECA rate, HPV positivity rates). The inclusion criteria were: (1) cervical smear testing performed at our institution during the study period and (2) cytology results available in the hospital information system. Exclusion criteria were: (1) being under 18 years of age, (2) missing demographic data, (3) duplicate records, and (4) samples of insufficient quality for interpretation.

### 2.3 Cervical cytology

Cervical samples were collected using the liquid-based cytology method. Experienced cytopathologists from the Department of Pathology at Haseki Training and Research Hospital evaluated all cytology samples. The results were reported according to the 2014 Bethesda System classification as follows: Negative for Intraepithelial Lesion or Malignancy (NILM), Atypical Squamous Cells of Undetermined Significance (ASC-US), Low-Grade Squamous Intraepithelial Lesion (LSIL), Atypical Squamous Cells, Cannot Exclude High-Grade Squamous Intraepithelial Lesion (ASC-H), High-Grade Squamous Intraepithelial Lesion (HSIL), Atypical Glandular Cells (AGC), Suspicion for Malignancy or Carcinoma. Epithelial cell abnormality (ECA) was defined as all cytology results other than NILM. This included ASC-US, LSIL, ASC-H, HSIL, AGC, and malignancy. Specimens reported as inadequate due to insufficient cellularity, confounding factors, or procedural artifacts were excluded but included in the overall statistics.

### 2.4 HPV testing

Our institution has used different HPV testing methods at different times during the course of our work. HPV testing was not a standard procedure between 2014–2019 and 2022–2023. Due to limited availability of test kits, HPV testing was performed selectively. It was not performed as routine screening but based on clinical indications (abnormal cytology, clinical suspicion, or patient request).

HPV mRNA Testing: During the 2020–2021 period, the Aptima HPV Assay (Hologic Inc., Marlborough, MA, USA) test was used for HPV screening. This test detects E6/E7 mRNA from 14 high-risk HPV types and provides reports for HPV-16, HPV-18/45, and other high-risk types (HPV-31, −33, −35, −39, −51, −52, −56, −58, −59, −66, −68).

HPV DNA Genotyping: The Bio-Speedy® Human Papillomavirus qPCR Kit (Bioeksen R&D Technologies Inc., Istanbul, Turkey; CE-IVD certified) was used for HPV DNA genotyping at our institution during 2024–2025. This assay detects 14 high-risk HPV genotypes (HPV-16, −18, −31, −33, −35, −39, −45, −51, −52, −56, −58, −59, −66, −68).

### 2.5 Histopathological evaluation

According to institutional protocols and ASCCP guidelines, colposcopy-directed cervical biopsies were performed on patients with abnormal cytology results. Experienced gynecologic pathologists evaluated the histopathological specimens. The results were grouped according to the terminology of cervical intraepithelial neoplasia (CIN): normal/benign, CIN grade 1 (CIN 1), CIN grade 2 (CIN 2), CIN grade 3 (CIN 3), adenocarcinoma *in situ* (AIS), and invasive carcinoma. For cytology-histopathology correlation analysis, only cases with cervical biopsy performed within 180 days of the index cytology were included. High-grade histologic lesions (CIN 2+) were defined as CIN 2, CIN 3, AIS, or invasive carcinoma.

### 2.6 Statistical analysis

Descriptive statistics were presented as mean ± standard deviation (SD), median, and interquartile range (IQR) or frequency and percentages, as appropriate. The chi-square (χ^2^) test or Fisher’s exact test was used for categorical variables. Spearman’s rank correlation coefficient (r) was used to assess correlations between continuous variables. Spearman correlation was used to analyze temporal trends and assess the direction and strength of change over time. The relationship between cytology results and HPV positivity was analyzed by calculating odds ratios (OR) with 95% confidence intervals (CI). The diagnostic performance of cervical cytology in detecting CIN 2 + lesions was assessed by calculating sensitivity, specificity, positive predictive value (PPV), and negative predictive value (NPV) with 95% CI using the Wilson score method. A two-tailed *p*-value below 0.05 was considered statistically significant. All statistical analyses were performed using SPSS version 26.0 (IBM Corp., Armonk, NY, USA) and Python 3.10 with the SciPy and Statsmodels libraries.

## 3 Results

**Figure 1:**
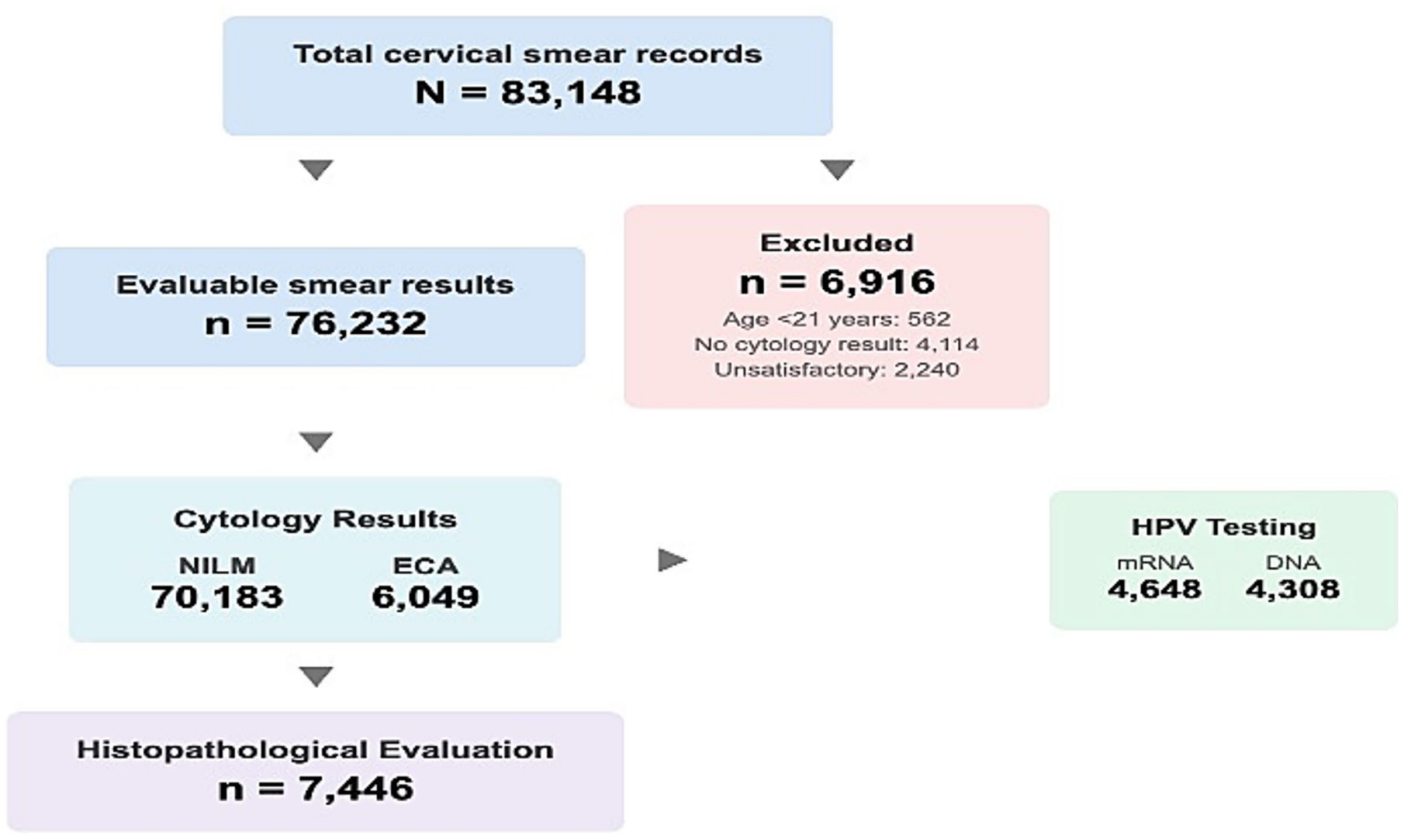
Flow chart of study population and screening pathway.

A total of 83,148 cervical smear records from January 2014 to December 2025 were identified. After excluding 6,916 records due to age <21 years (*n* = 562), missing cytology results (*n* = 4,114), or unsatisfactory specimens (*n* = 2,240), 76,232 evaluable cervical smear results were included in the final analysis (Figure 1). HPV testing was performed in two distinct periods: HPV mRNA testing (Aptima assay) during 2020–2021 (*n* = 4,648) and HPV DNA genotyping during 2024–2025 (*n* = 4,308). Histopathological evaluation was available for 7,446 women who underwent cervical biopsy.

### 3.1 Study population and demographics

A total of 83,148 cervical smear records were identified over the 12-year study period (January 2014 to December 2025). After excluding 562 patients aged <21 years, 82,586 smear tests from 66,684 unique patients were included in the main analysis. The mean age was 42.2 ± 11.5 years (median: 42 years; interquartile range: 33–49 years). The majority of patients (47.6%) were between 35 and 49 years of age. HPV mRNA testing (Aptima HPV Assay, Hologic Inc.) was performed in 4,648 patients during 2020–2021, HPV DNA genotyping was performed in 4,308 cases during 2024–2025, and histopathological evaluation was available for 7,446 women who underwent cervical biopsy during the study period (Table 1).

**Table 1:** Demographic and clinical characteristics of the study population.

Characteristic	*n* (%)	Mean ± SD/Median (IQR)
Total smear records	83,148	—
Patients aged <21 years (excluded)	562	—
Smear tests (≥21 years)	82,586	—
Unique patients (≥21 years)	66,684	—
Age (years)	—	42.2 ± 11.5/42 (33–49)
HPV mRNA testing	4,648	—
HPV DNA testing	4,308	—
Biopsy performed	7,446	—

SD, Standard deviation; IQR, Interquartile range; HPV, Human papillomavirus.

### 3.2 Distribution of cytology results

**Figure 2:**
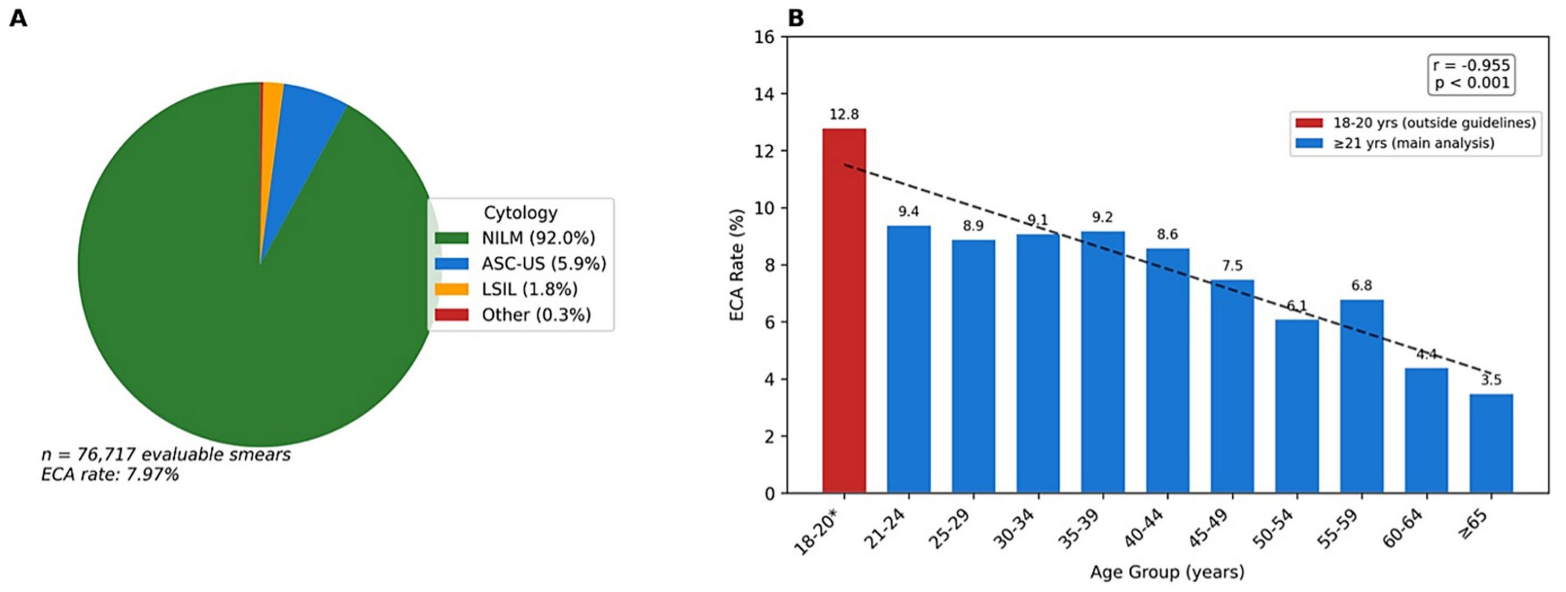
Distribution of cervical cytology results **(A)** and age-specific ECA rates **(B)**.

Among 82,586 smear tests from patients aged ≥21 years, 6,354 were excluded due to missing cytology results (*n* = 4,114) or unsatisfactory specimens (*n* = 2,240), leaving 76,232 evaluable results. Of these, 70,183 (92.07%) were classified as negative for intraepithelial lesion or malignancy (NILM). The overall epithelial cell abnormality (ECA) rate was 7.93% (*n* = 6,049). ASC-US was the most common abnormality (*n* = 4,443; 5.83%), followed by LSIL (*n* = 1,347; 1.77%), HSIL (*n* = 128; 0.17%), ASC-H (*n* = 94; 0.12%), AGC (*n* = 27; 0.04%), and malignancy (*n* = 10; 0.01%) (Table 2; Figure 2A).

**Table 2:** Distribution of cervical cytology results according to Bethesda 2014 classification.

Cytology category	*n*	% (Evaluable)
NILM	70,183	92.07
ASC-US	4,443	5.83
LSIL	1,347	1.77
HSIL	128	0.17
ASC-H	94	0.12
AGC	27	0.04
Malignancy	10	0.01
ECA Total	**6,049**	**7.93**
Evaluable Total	**76,232**	**100.0**

Excluded: No cytology result (*n* = 4,114) and unsatisfactory specimens (*n* = 2,240).

NILM, Negative for intraepithelial lesion or malignancy; ASC-US, Atypical squamous cells of undetermined significance; LSIL, Low-grade squamous intraepithelial lesion; ASC-H, Atypical squamous cells, cannot exclude HSIL; HSIL, High-grade squamous intraepithelial lesion; AGC, Atypical glandular cells; ECA, Epithelial cell abnormality. Bold values indicate summary/total rows or key aggregate values to distinguish them from individual category data.

### 3.3 Age-stratified analysis

ECA rates varied significantly across age groups (χ^2^ = 264.70, df = 10, *p* < 0.001). The highest rate was observed in the 18–20 age group (12.78%), declining to 3.48% in patients aged ≥65 years (Spearman r = −0.955, *p* < 0.001). The 18–20 age group (*n* = 485) had an ECA rate of 12.78% compared to 3.48–9.36% in older age groups (Table 3; Figure 2B).

**Table 3:** Epithelial cell abnormality rates according to age groups.

Age group	Total (*n*)	ECA (*n*)	ECA rate (%)	95% CI
18–20	485	62	12.78	10.10–16.05
21–24	3,684	345	9.36	8.47–10.35
25–29	8,483	756	8.91	8.32–9.54
30–34	9,550	871	9.12	8.56–9.71
35–39	11,044	1,021	9.24	8.72–9.80
40–44	12,964	1,115	8.60	8.13–9.10
45–49	12,264	924	7.53	7.08–8.01
50–54	8,314	503	6.05	5.56–6.58
55–59	4,320	295	6.83	6.11–7.62
60–64	2,709	118	4.36	3.65–5.19
≥65	2,900	101	3.48	2.87–4.21
Total	**76,717**	**6,111**	**7.97**	—

ECA, Epithelial cell abnormality (ASC-US or worse); CI, Confidence interval. Table includes 18–20 age group (*n* = 485) for comparative purposes; primary outcome calculations (overall ECA rate: 7.93%) are based on ≥21 years population (*n* = 76,232). Chi-square test: χ^2^ = 264.70, df = 10, *p* < 0.001; Spearman correlation: r = −0.955, *p* < 0.001. Bold values indicate summary/total rows or key aggregate values to distinguish them from individual category data.

### 3.4 HPV testing results

#### 3.4.1 HPV mRNA testing

**Figure 3:**
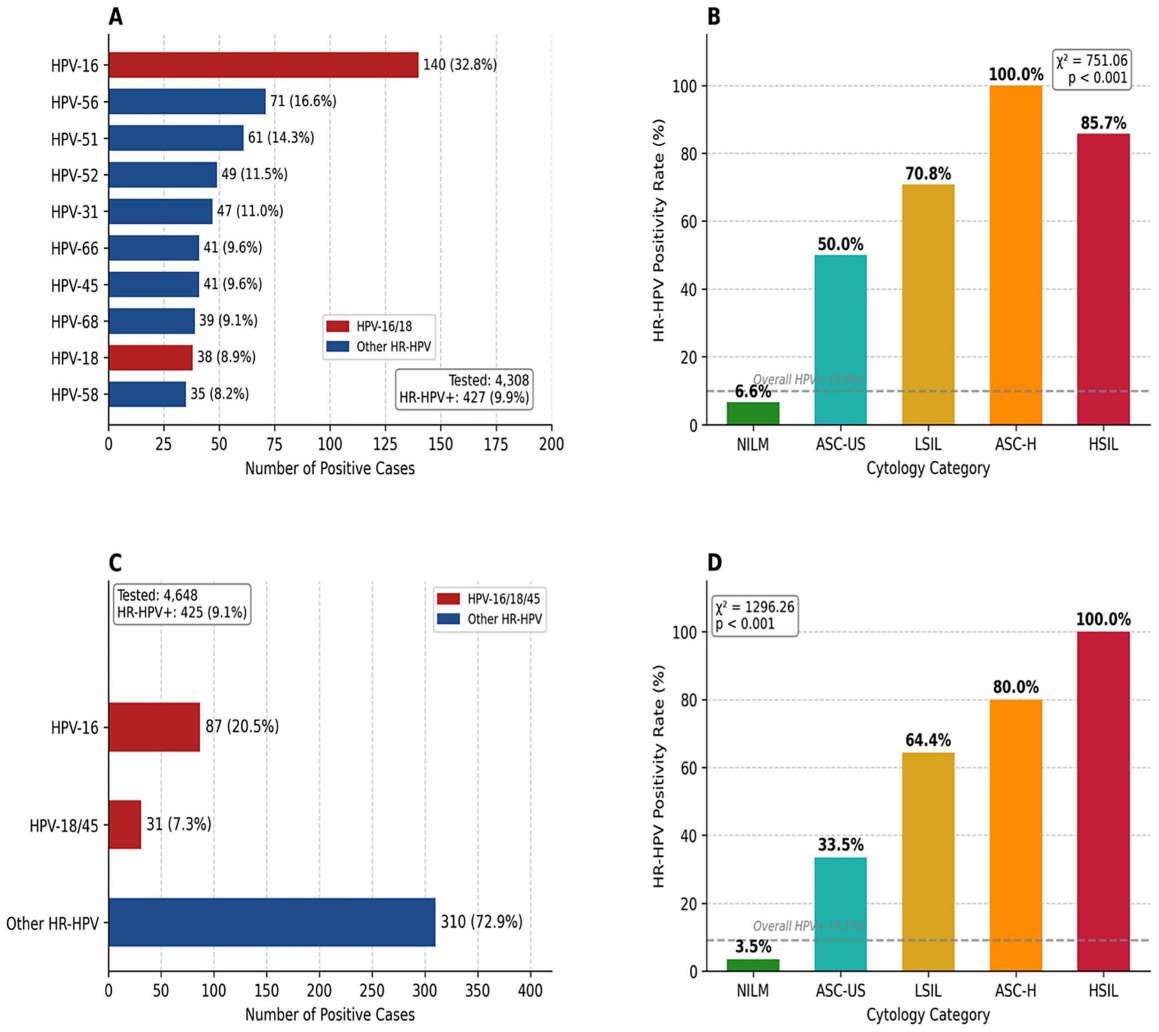
High-risk HPV genotype distribution and cytology correlation. **(A)** HPV DNA genotyping (*n* = 4,308). HR-HPV positivity: 9.9% (*n* = 427). **(B)** HR-HPV DNA positivity rates by cytology category (χ^2^ = 751.06, *p* < 0.001). **(C)** HPV mRNA testing (Aptima, *n* = 4,648). HR-HPV positivity: 9.1% (*n* = 425). **(D)** HR-HPV mRNA positivity rates by cytology category (χ^2^ = 1296.26, *p* < 0.001). Dashed lin es in dicate overall HPV positivity rates.

HPV mRNA testing using the Aptima HPV Assay was performed on 4,648 samples during 2020–2021. High-risk HPV mRNA was detected in 425 cases (9.1%; 95% CI: 8.3–10.0%) (Table 4; Figure 3C).

**Table 4:** HPV mRNA (Aptima) testing results (2020–2021).

Result	*n*	%	95% CI
Total tested	4,648	100.0	—
HR-HPV Negative	4,223	90.9	90.0–91.7
HR-HPV Positive	425	9.1	8.3–10.0
HPV-16 positive	87	20.5*	16.9–24.6
HPV-18/45 positive	31	7.3*	5.2–10.2
Other HR-HPV	310	72.9*	68.5–76.9

HR-HPV, High-risk human papillomavirus; CI, Confidence interval. HR-HPV Negative, Tested negative for the 14 high-risk HPV genotypes (HPV-16, -18, -31, -33, -35, -39, -45, -51, -52, -56, -58, -59, -66, -68). *Percentage of HR-HPV positive cases. Three patients had HPV-16 and HPV-18/45 co-infection; individual genotype counts exceed total due to co-infections.

Among HR-HPV positive cases, genotype-specific analysis revealed HPV-16 in 87 cases (20.5% of HR-HPV positive), HPV-18/45 in 31 cases (7.3%), and other high-risk types (non-16/18/45) in 310 cases (72.9%).

#### 3.4.2 HPV DNA genotyping

Among 4,308 women who underwent HPV DNA genotyping, 427 (9.9%) were positive for at least one high-risk HPV genotype. HPV-16 was the most frequently detected genotype, identified in 140 cases (32.8% of HR-HPV positive), followed by HPV-56 (*n* = 71, 16.6%), HPV-51 (*n* = 61, 14.3%), HPV-52 (*n* = 49, 11.5%), and HPV-31 (*n* = 47, 11.0%). HPV-18 was detected in 38 cases (8.9%). Combined, HPV-16 and/or HPV-18 were present in 171 cases (40.0% of HR-HPV positive), while other high-risk genotypes accounted for 60.0% of infections (Table 5; Figure 3A).

**Table 5:** Distribution of high-risk HPV genotypes by DNA testing (2024–2025).

HPV genotype	*n*	%*
HPV-16	140	32.8
HPV-56	71	16.6
HPV-51	61	14.3
HPV-52	49	11.5
HPV-31	47	11.0
HPV-45	41	9.6
HPV-66	41	9.6
HPV-68	39	9.1
HPV-18	38	8.9
HPV-58	35	8.2
HPV-59	30	7.0
HPV-39	29	6.8
HPV-35	23	5.4
HPV-33	20	4.7

HPV, Human papillomavirus; HR-HPV, High-risk HPV. Total tested: 4,308; HR-HPV positive: 427 (9.9%). *Percentage of HR-HPV positive cases (*n* = 427). Percentages exceed 100% due to multiple genotype co-infections in some patients.

### 3.5 Correlation between cytology and HPV status

#### 3.5.1 Cytology-HPV mRNA correlation

HR-HPV mRNA positivity rates increased significantly with cytological abnormality severity. Among 3,956 NILM cases, 3.5% were HR-HPV positive. Positivity rates increased to 33.5% (141/421) in ASC-US, 64.4% (121/188) in LSIL, 80.0% (4/5) in ASC-H, and 100% (12/12) in HSIL cases (Table 6; Figure 3D). Compared to NILM, the odds of HR-HPV positivity were significantly elevated for ASC-US (OR 13.83, 95% CI 10.62–18.00), LSIL (OR 49.59, 95% CI 35.19–69.89), and ASC-H (OR 109.84, 95% CI 12.20–989.22; all *p* < 0.001). Chi-square analysis confirmed a highly significant association between cytology category and HPV mRNA status (χ^2^ = 1296.26, df = 4, *p* < 0.001).

**Table 6:** HR-HPV mRNA positivity according to cytology category (2020–2021).

Cytology	Total (*n*)	HPV+ (*n*)	HPV+ (%)	95% CI	OR (95% CI)	*p*-value
NILM	3,956	139	3.5	3.0–4.1	1.00 (Reference)	—
ASC-US	421	141	33.5	29.2–38.1	13.83 (10.62–18.00)	<0.001
LSIL	188	121	64.4	57.3–70.9	49.59 (35.19–69.89)	<0.001
ASC-H	5	4	80.0	37.6–96.4	109.84(12.20–989.22)	<0.001
HSIL	12	12	100.0	75.7–100.0	∞	<0.001
Total	**4,582**	**417**	**9.1**	**8.3–10.0**	—	—

HR-HPV, High-risk human papillomavirus; CI, Confidence interval; OR, Odds ratio. Chi-square: χ^2^ = 1296.26, df = 4, *p* < 0.001. Excludes cases with unsatisfactory cytology (*n* = 64) and AGC (*n* = 2). Bold values indicate summary/total rows or key aggregate values to distinguish them from individual category data.

#### 3.5.2 Cytology-HPV DNA correlation

Similarly, HR-HPV DNA positivity demonstrated a strong correlation with cytological abnormalities. Among 3,708 NILM cases, 6.6% were HR-HPV positive. Positivity rates increased to 50.0% (98/196) in ASC-US, 70.8% (51/72) in LSIL, 100% (2/2) in ASC-H, and 85.7% (6/7) in HSIL cases (Table 7; Figure 3B). Compared to NILM, the odds of HR-HPV positivity were significantly elevated for ASC-US (OR 14.20, 95% CI 10.43–19.33; *p* < 0.001), LSIL (OR 34.48, 95% CI 20.41–58.25; *p* < 0.001), and HSIL (OR 85.18, 95% CI 10.21–710.37; *p* < 0.001). Chi-square analysis confirmed a significant association between cytology category and HPV DNA status (χ^2^ = 751.06, df = 4, *p* < 0.001).

**Table 7:** HR-HPV DNA positivity according to cytology category (2024–2025).

Cytology	Total (*n*)	HPV+ (*n*)	HPV+ (%)	95% CI	OR (95% CI)	*p*-value
NILM	3,708	244	6.6	5.8–7.4	1.00 (Reference)	—
ASC-US	196	98	50.0	43.1–56.9	14.20 (10.43–19.33)	<0.001
LSIL	72	51	70.8	59.5–80.1	34.48 (20.41–58.25)	<0.001
ASC-H	2	2	100.0	34.2–100.0	∞	0.004
HSIL	7	6	85.7	48.7–97.4	85.18 (10.21–710.37)	<0.001
Total	**3,985**	**401**	**10.1**	**9.2–11.0**	—	—

HR-HPV, High-risk human papillomavirus; CI, Confidence interval; OR, Odds ratio. Chi-square: χ^2^ = 751.06, df = 4, *p* < 0.001. Excludes cases with no cytology result (*n* = 301) and unsatisfactory cytology (*n* = 22).

#### 3.5.3 HPV-histopathology correlation

Among 146 biopsy specimens from women who underwent both HPV DNA testing and cervical biopsy, HR-HPV positive women demonstrated significantly higher rates of high-grade histopathological lesions compared to women who tested negative for high-risk HPV genotypes. CIN2 + was detected in 47.1% (16/34) of HR-HPV positive cases compared to 16.1% (18/112) of cases that tested negative for the 14 high-risk HPV genotypes (OR 4.64, 95% CI 2.00–10.77; χ^2^ = 12.34, *p* < 0.001). Among HR-HPV positive women, CIN2 + rates were 25.0% (3/12) for HPV-16/18 and 59.1% (13/22) for other HR-HPV genotypes (Table 8).

**Table 8:** HPV status and histopathological outcomes.

HPV status	Biopsy (*n*)	CIN2 + (*n*)	CIN2 + (%)	95% CI	OR (95% CI)	*p*-value
HR-HPV Negative	112	18	16.1	10.4–24.0	1.00 (Ref.)	—
HR-HPV Positive	34	16	47.1	31.5–63.3	4.64(2.00–10.77)	<0.001
HPV-16/18	12	3	25.0	8.9–53.2	—	—
Other HR-HPV	22	13	59.1	38.7–76.7	—	—
Total	**146**	**34**	**23.3**	—	—	—

HR-HPV, High-risk human papillomavirus; CIN2+, Cervical intraepithelial neoplasia grade 2 or worse; CI, Confidence interval; OR: Odds ratio. HR-HPV Negative, Tested negative for the 14 high-risk HPV genotypes. Chi-square: χ^2^ = 12.34, df = 1, *p* < 0.001. Biopsy performed in women with HPV DNA testing (2024–2025). Bold values indicate summary/total rows or key aggregate values to distinguish them from individual category data.

### 3.6 Histopathological outcomes

A total of 2,039 cervical biopsies with evaluable histopathological results were identified among patients aged ≥21 years during the study period. The distribution of histopathological diagnoses is presented in Table 9. CIN 1 was the most common diagnosis (*n* = 1,080, 53.0%), followed by CIN 3 (*n* = 437, 21.4%), CIN 2 (*n* = 301, 14.8%), adenocarcinoma (*n* = 142, 7.0%), and invasive squamous cell carcinoma (*n* = 77, 3.8%). High-grade lesions (CIN 2+) were identified in 959 cases (47.0%).

**Table 9:** Distribution of histopathological diagnoses among cervical biopsies.

Histopathology	*n*	Percentage (%)
CIN 1	1,080	52.9
CIN 2	301	14.8
CIN 3	437	21.4
Adenocarcinoma	142	7.0
Invasive squamous cell carcinoma	77	3.8
AIS	2	0.1
Total	**2,039**	**100.0**
CIN 2 + (High-grade)	**959**	**47.0**

CIN, Cervical intraepithelial neoplasia; AIS, Adenocarcinoma in situ. Cervical biopsies from patients aged ≥21 years with evaluable histopathology results (2014–2025). Includes only preneoplastic and neoplastic lesions. Excludes normal/benign (*n* = 5,126), other diagnoses (*n* = 220), and unsatisfactory specimens (*n* = 61). Bold values indicate summary/total rows or key aggregate values to distinguish them from individual category data.

### 3.7 Cytology-histopathology correlation

Among 2,119 patients with matched cytology-histopathology results (biopsy within 180 days of cytology), the mean interval was 45.8 days. The positive predictive value (PPV) for CIN 2 + increased with cytological abnormality severity: 4.5% for NILM, 8.0% for ASC-US, 21.6% for LSIL, 29.6% for ASC-H, and 89.2% for HSIL (Table 10).

**Table 10:** Cytology-histopathology correlation and CIN 2 + detection rates.

Cytology	Biopsy (*n*)	CIN2 + (*n*)	PPV (%)	95% CI
NILM	1,297	59	4.5	3.5–5.8
ASC-US	501	40	8.0	5.9–10.7
LSIL	255	55	21.6	17.0–27.0
ASC-H	27	8	29.6	15.9–48.5
HSIL	37	33	89.2	75.3–95.7
Malignancy	2	2	100.0	34.2–100.0

CIN, Cervical intraepithelial neoplasia; PPV, Positive predictive value; CI, Confidence interval. Biopsy performed within 180 days of cytology (*n* = 2,119). Excludes cases with no cytology result (*n* = 136), unsatisfactory cytology (*n* = 95), and AGC (*n* = 9).

Using the ≥ASC-US threshold for CIN 2 + detection, sensitivity was 70.1%, specificity was 64.4%, PPV was 16.8%, and NPV was 95.5% (Table 11).

**Table 11:** Diagnostic performance of cervical cytology for CIN 2 + detection at different thresholds.

Threshold	Sensitivity (%)	Specificity (%)	PPV (%)	NPV (%)	Accuracy (%)
≥ASC-US	70.1	64.4	16.8	95.5	64.9
≥LSIL	49.7	88.4	30.5	94.5	84.8
≥ASC-H	21.8	98.8	65.2	92.5	91.6
≥HSIL	17.8	99.8	89.7	92.2	92.2

PPV, Positive predictive value; NPV, Negative predictive value. Target condition: CIN 2 or worse (CIN 2+).

### 3.8 Temporal trends analysis

**Figure 4:**
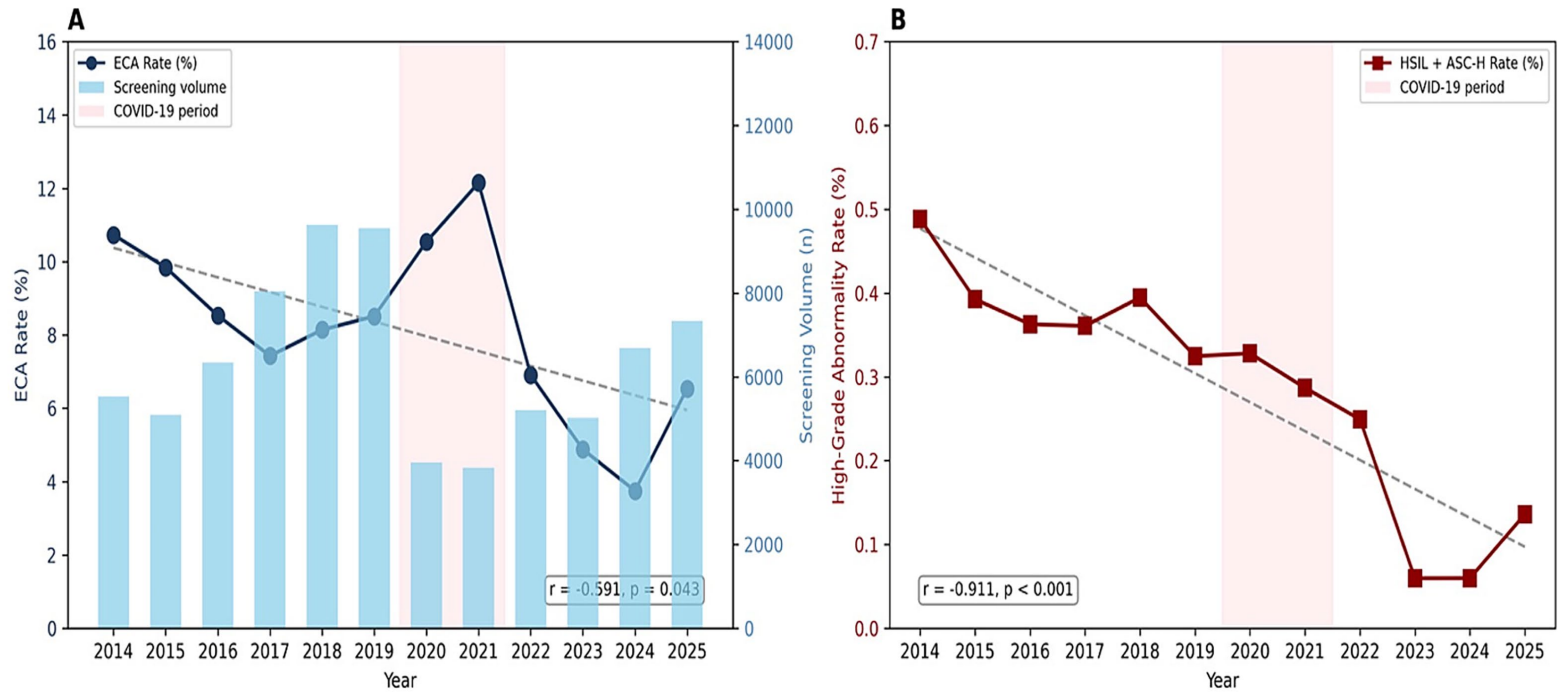
Temporal trends in cervical cancer screening (2014–2025). **(A)** Annual epithelial cell abnormality (ECA) rates (blue line) and screening volumes (light blue bars). A declining trend in ECA rates was observed (*r* = −0.591, *p* = 0.043). **(B)** There is a significant declining trend in high-grade abnormality rates (HSIL + ASC-H) (*r* = −0.911, *p* < 0.001). The pink shading indicates the period of the pandemic (2020–2021).

Annual screening volumes and ECA rates from 2014 to 2025 are presented in Table 12 and Figure 4A. Linear trend analysis showed a statistically significant declining ECA rate over the study period (slope = −0.40%/year, R^2^ = 0.349, *p* = 0.043). The rate of high-grade abnormalities (HSIL + ASC-H) demonstrated an even more pronounced decline, decreasing from 0.49% (27/5,527) in 2014 to 0.14% (10/7,341) in 2025 (r = −0.911, *p* < 0.001) (Figure 4B).

**Table 12:** Annual screening volumes and epithelial cell abnormality rates.

Year	Total (*n*)	ECA (*n*)	ECA Rate (%)	95% CI
2014	5,527	593	10.73	9.94–11.57
2015	5,094	501	9.84	9.05–10.68
2016	6,339	541	8.53	7.87–9.25
2017	8,037	597	7.43	6.87–8.02
2018	9,629	784	8.14	7.61–8.71
2019	9,548	813	8.51	7.97–9.09
2020*	3,964	418	10.54	9.63–11.54
2021*	3,834	466	12.15	11.16–13.23
2022	5,212	360	6.91	6.25–7.63
2023	5,021	245	4.88	4.32–5.51
2024	6,686	251	3.75	3.32–4.24
2025	7,341	480	6.54	6.00–7.13
Total	**76,232**	**6,049**	**7.93**	—

ECA, Epithelial cell abnormality; CI, Confidence interval. *COVID-19 pandemic period. Bold values indicate summary/total rows or key aggregate values to distinguish them from individual category data.

Mean annual screening volume was 7,362 during the pre-pandemic period (2014–2019), decreasing to 3,899 during 2020–2021 (47.0% reduction), and partially recovering to 6,065 during 2022–2025 (Figure 4B). The ECA rate was 8.67% pre-pandemic, 11.34% during 2020–2021 (χ^2^ = 56.90, *p* < 0.001), and 5.51% post-pandemic.

## 4 Discussion

In our study, we retrospectively evaluated a total of 83,148 cervical smear samples from women who visited a tertiary hospital in Istanbul, Turkey. The overall epithelial cell abnormality (ECA) rate was 7.93% among the 76,232 evaluable samples obtained from women aged 21 years and older. The most frequently detected abnormality was ASC-US (5.83%), followed by LSIL (1.77%), HSIL (0.17%), and ASC-H (0.12%). The HPV test was performed using two different methods in two separate periods: the HPV mRNA test (Aptima) performed on 4,648 samples in 2020–2021 showed a positivity rate of 9.1%, while the HPV DNA genotyping test performed on 4,308 samples in the 2024–2025 period showed a positivity rate of 9.9%. HPV-16 was the most prevalent genotype in both methods, accounting for 20.5% of HPV mRNA positive cases and 32.8% of HPV DNA positive cases. A high correlation was found between cytology and histopathology, and the positive predictive value (PPV) of HSIL in predicting CIN2 + was evaluated at 89.2%. Additionally, a significant decrease in ECA rates was found over the 12-year period, suggesting improvements in the effectiveness and awareness of cervical screening.

The rate of ECA in this study was 7.93%, which is similar to the rates reported in other medical centers in Turkey. These rates generally range from 5 to 15%. However, this rate is higher than the 2 to 5% rates reported in community-based screening programs conducted in developed countries. This difference may be due to referral bias, since hospital-based studies tend to focus on patients with symptoms and individuals with prior abnormalities (21). A multicenter study conducted by the Turkish Cervical Cancer and Cervical Cytology Research Group reported that ECA rates in different regions of Turkey ranged from 1.8 to 6.8%. These differences are associated with variations in demographic structure, screening procedures, and laboratory evaluation standards (22).

The 5.83% ASC-US rate in our study was within the 2–10% range reported in the literature. However, differences may be seen between laboratories due to variations in cytological reporting standards (17, 23). The HSIL rate was determined to be 0.17%, which is close to the lower limit of the 0.1–2.0% range reported from other tertiary centers in Turkey and may be related to our institution primarily serving a screening-based population (24). The 2019 ASCCP Risk-Based Management Consensus Guidelines emphasize that institutional ECA rates should be considered in the development of clinical management algorithms, as these rates directly affect the positive predictive value of cytological findings for high-grade disease (25).

HPV positivity rates in our study were found to be 9.1% for mRNA testing and 9.9% for DNA genotyping; these rates are higher than the 3.5% rate reported in the Turkish national community-based screening program (13). This situation may be attributed to the selection of higher-risk women in the hospital setting. HPV positivity rates in hospital-based studies reported from different centers in Turkey range from 14.2 to 36.3%; the rates in our study are in the lower part of this range, which may be due to the combined evaluation of routine screening patients and symptomatic referrals (26–28). In a multicenter study by the Turkish Gynecologic Oncology Group involving 6,388 patients, the overall HPV positivity rate was reported as 25%, and significant regional differences were demonstrated (28). It should be noted that the two HPV testing methods used in our study differ in their analytical targets: the Aptima assay detects E6/E7 mRNA expression, a marker of active oncogenic transcription, while the Bio-Speedy qPCR kit detects HPV DNA regardless of transcriptional activity. The selection of testing platforms was not a deliberate methodological choice by the investigators but was determined by institutional procurement during each respective period. Despite these differences, overall HR-HPV positivity rates were similar (9.1% vs. 9.9%). However, genotype-level comparison between the two periods is limited, as the Aptima assay reports results in three grouped categories (HPV-16, HPV-18/45, and other HR-HPV) rather than providing individual genotype identification. Previous studies have demonstrated that mRNA-based assays may yield different positivity profiles compared to DNA-based methods due to their selective detection of transcriptionally active infections (20). Therefore, the results from the two testing periods should be interpreted independently rather than as a direct methodological comparison.

In our study, HPV-16 was the predominant genotype, present in 32.8% of DNA samples and 20.5% of mRNA samples. This finding is similar to other data indicating that HPV-16 is the most common type of HPV in cervical cancer and its precursors (9). However, our rate was lower than the reported rate of 61.7% for invasive cervical cancers; this may be because the study population included not only cancer patients but also a larger spectrum of diseases. HPV-56 (16.6%) and HPV-51 (14.3%) were the second and third most common, respectively, a distribution that differed from the global pattern where HPV-18 is usually second. Our high-risk genotype distribution is consistent with data from the Middle East and North Africa region, where types other than 16/18 contribute significantly (29, 30). These findings are important for vaccination strategies. Although the current nonvalent vaccines covering HPV-16, -18, -31, -33, -45, -52, and -58 protect against many high-risk types in our population, they do not protect against HPV-56 and HPV-51, which were frequently observed in our study (31).

Cytology-histopathology correlation has shown the high diagnostic performance of cervical cytology at our center. The PPV of 89.2% for HSIL cytology in CIN2 + prediction is at the upper limit of the 70–90% range reported in meta-analyses and confirms the high diagnostic accuracy of HSIL cytology (32, 33). ASCCP guidelines recommend treatment before biopsy in patients with a CIN3 + risk above 60%, HSIL cytology, and HPV-16 positivity, highlighting the clinical importance of this category (25). The CIN2 + rate in the ASC-H group was 29.6%, which is consistent with the 25–50% range reported in the literature and supports the recommendation for colposcopy in all ASC-H (34). The progression rate from LSIL to CIN2 + was 21.6%, which is consistent with the spontaneously regressing nature of most low-grade lesions, particularly in young women (35, 36).

Using the ≥ASC-US cutoff point, the sensitivity (70.1%) and specificity (64.4%) for detecting CIN2 + were high, supporting the importance of cytology as a triage tool in HPV-based screening algorithms (37). The ALTS study has shown that HPV testing provides superior risk classification compared to repeat cytology in borderline cytology results; this finding supports the HPV-based screening and cytology triage approach implemented in Turkey (38). Although other high-risk genotypes showed higher CIN2 + rates than HPV-16/18 (59.1% vs. 25.0%), this finding should be viewed with caution due to the small sample size. While the total number of cases included in the HPV-histopathology correlation analysis was 146, this number was only 12 for HPV-16/18. This unexpected result may be due to the selective application of clinically indicated HPV testing due to limited availability of kits. Cases in which genotypes other than 16/18 were detected and referred for biopsy may represent a higher-risk subgroup with more pronounced cytological abnormalities. Furthermore, the small sample size reduces statistical power. Prospective studies are needed to determine more clearly the relationship between specific HPV genotypes and histopathological findings in the Turkish population.

The downward trend observed in ECA rates over the 12-year follow-up period (from 10.73% in 2014 to 6.54% in 2025; *r* = −0.591, *p* = 0.043) may be due to several factors, including improved access to healthcare, increased public education, expanded screening, and improved cytology laboratory standards (39). The more significant decrease in high-grade abnormalities (HSIL + ASC-H) from 0.49% in 2014 to 0.14% in 2025 (*r* = −0.911, *p* < 0.001) shows that precancerous lesions are being identified and treated more effectively. Although the HPV vaccine has not yet been included in Turkey’s national immunization program, voluntary vaccination programs may have contributed to the trends observed, particularly in younger age groups (40). Data from countries that have implemented national HPV vaccination programs for many years, such as Sweden and the United Kingdom, show significant declines in both cervical abnormalities and invasive cancer incidence; moreover, it has been proven that administering the vaccine before the first sexual intercourse increases its effectiveness (41, 42). Follow-up data spanning up to fourteen years have shown that the vaccine provides lasting protection against HPV-16/18 infection and associated precancerous lesions (43). The WHO’s Global Strategy to Accelerate Cervical Cancer Elimination provides a clear plan for eliminating the disease through vaccination (90%), screening (70%), and treatment (90%) (5). Our study shows that Turkey is progressing toward these targets. However, continuous efforts are required to increase vaccination rates and maintain high screening participation.

Our study has many strengths. The twelve-year period represents one of the longest trends in cervical cytology analysis obtained from a single center. In addition, temporal changes have been reliably assessed. The large sample size of 83,148 smears provided sufficient statistical power to detect meaningful differences between subgroups and time periods. The consistent utilization of the Bethesda System and protocols throughout the study minimized classification differences. Furthermore, the heterogeneous patient population served by our tertiary institution, located in Istanbul and receiving referrals from a large metropolitan area and surrounding regions, increases the generalizability of the findings to similar urban Turkish populations. However, certain limitations should be considered. Retrospective design prevents prospective validation of diagnostic accuracy and limits control of potential confounding variables. Because this was a hospital-based study, our results may not be directly generalizable to community-based screening programs due to referral bias. The HPV test was only introduced in 2020, limiting the sample size for HPV-related analyses (*n* = 4,648 for mRNA; *n* = 4,308 for DNA genotyping). Furthermore, the selective application of HPV testing based on clinical indication may have led to a higher-risk population being tested and may partially explain the unexpectedly high CIN2 + rates observed in non-16/18 genotypes (59.1% vs. 25.0%). The fact that histopathological follow-up was not completed in all cases with abnormal cytology results raises the possibility that some high-grade lesions may have been missed. The limited size of the HPV-histopathology correlation analysis (n = 146) also reduced its statistical power. Furthermore, due to the absence of data on potential confounding variables such as socioeconomic status, parity, smoking history, and sexual behavior, vaccination status, and marital status in the electronic records, these factors could not be included in the analyses. Future prospective studies should incorporate these variables to enable a more comprehensive analysis of factors influencing cervical cytological abnormalities and HPV prevalence.

In conclusion, our study, conducted at a tertiary care center, provides comprehensive epidemiological data that contributes to cervical cancer prevention strategies. The ECA rate identified as 7.93%, HPV positivity rates ranging between 9.1 and 9.9%, and HPV-16 being the most common type in positive cases are all consistent with data from the same region. These findings clearly demonstrate the importance of continuing screening programs without disruption. Future vaccination policies should consider the high prevalence of non-vaccine-targeted genotypes, such as HPV-56 and HPV-51. The observed decrease in cytological abnormalities is a positive sign that may be attributed to increased screening coverage, improved public health awareness, and the early effects of vaccination. The strong correlation between cytology and histopathology of HSIL, with a PPV of 89.2% for CIN2+, confirms the reliability of cervical cytology when performed according to standard protocols. Our findings support the current national screening strategy that includes HPV testing and cytology screening, and they highlight the need for continued investment in cervical cancer prevention programs. Regular monitoring of cervical cytology and HPV data and updating prevention strategies according to the changing structure of society are of great importance for Turkey to achieve the World Health Organization’s elimination targets.

## Data Availability

The datasets used and/or analyzed during the current study are not publicly available due to patient confidentiality and ethical restrictions but may be made available from the corresponding author upon reasonable request and upon obtaining additional ethics committee approval.
